# Identification of miRNAs Differentially Expressed in Clinical Stages of Human Colorectal Carcinoma—An Investigation in Guangzhou, China

**DOI:** 10.1371/journal.pone.0094060

**Published:** 2014-04-17

**Authors:** Xue-Hu Xu, Xiao-Bing Wu, Shang-Biao Wu, Hai-Bo Liu, Rong Chen, Yong Li

**Affiliations:** 1 Gastrointestinal Surgery, Third Affiliated Hospital of Guangzhou Medical University, Guangzhou, China; 2 Department of Experimental Research, Third Affiliated Hospital of Guangzhou Medical University, Guangzhou, China; University of North Carolina School of Medicine, United States of America

## Abstract

Aberrant expression of microRNAs (miRNAs) has been implicated in human cancer, including colorectal cancer (CRC). Such dysregulated miRNAs may have potential as diagnostic markers or therapeutic targets. However, the nature of an association between these miRNAs and clinical stages of CRC is still not clear. To this end, we performed a miRNA profiling of 1547 distinct human miRNAs using 31 samples of tumor and paired normal mucosa obtained from 31 CRC patients. Based on statistical analyses of profiling data, we identified 569 miRNAs that were significantly dysregulated in CRC relative to normal tissues (*P*<0.05). Among the 569 dysregulated miRNAs, downregulation of 17 was associated with stages II, III, and IV colon and rectal cancers (separate or combined), according to our criteria. We also assessed the potential of these dysregulated miRNAs as diagnostic biomarkers for CRC patients who were without metastasis, and the value of the dysregulated miRNAs for predicting metastasis, lymph node and distant. Their distinct expression patterns in colon and rectal cancers were also examined. Although our findings cannot be immediately applied toward clinical diagnosis, our new study model for determining and assessing the biomarker potential of dysregulated miRNAs should be useful in further research in detection of human CRC.

## Introduction

Colorectal cancer (CRC) is a serious problem for human health [1]. The incidence and mortality of CRC in China has increased rapidly in the past few decades [2]. Early detection is essential to reduce mortality and improve survival rates, but is hampered by the lack of convenient screening tools with high specificity and sensitivity for early-stage tumors. Therefore, novel biomarkers for detection of early-stage CRC are urgently required. The identification of aberrantly expressed microRNAs (miRNAs) specific to CRC is a new approach that may lead to the development of clinical biomarkers for cancer screening and early detection.

MiRNAs are a class of small noncoding RNAs that regulate gene expression at the post-transcriptional level, mainly by binding to the 3′-untranslated regions (UTRs) of target mRNAs, leading to mRNA degradation or inhibition of translation [3]. Since the first miRNA was discovered in *Caenorhabditis elegans* in the early 1990s [4], many miRNAs have been found in plants and animals. A total of 2578 human miRNAs sequences have been collected in the most recent version of miRBase (V20). It has been predicted that about one-third of human genes are regulated by miRNAs [5]. An aberrant miRNA expression signature is a hallmark of several diseases, including human cancer [6]–[8]. The importance of miRNA in cancer is highlighted by the observation that about 50% of miRNAs are located in cancer-associated genomic regions or fragile sites, which are frequently amplified or deleted during tumorigenesis [9].

The report of Piepoli et al. [10] indicated that miRNAs may be used as diagnostic biomarkers for tissue-specific cancers, including CRC. For example, Frerichs et al. [11] reported that miR-196a is pro-oncogenic in CRC, while Bandrés et al. [1] suggested that a miRNA expression profile could be relevant to the understanding of the biological and clinical behaviors of colorectal neoplasia. The association of miRNAs with tumorigenesis has highlighted their potential as diagnostic markers and therapeutic targets in CRC [12].

However, several issues in miRNA studies of CRC remain unresolved, including the pattern or signature of differentially expressed miRNAs associated with specific clinical stages. We would like to emphasize the importance of identifying the characteristic differential expressions of miRNAs in different tumor stages. While previous studies on dysregulated miRNAs have focused on comparisons between cancer patients and normal controls, there has been little study of the expression patterns in each stage of tumor. In addition, analyzed samples are sometimes obtained only from colon or rectal cancer, but verified diagnostic markers of CRC should be able to distinguish CRC patients in all stages from normal subjects. This might prevent the misdiagnosis of patients with certain stages of CRC. Furthermore, ideally the expression patterns of miRNAs used as diagnostic markers in patients without lymph node or distant metastasis should differ from those of patients in other stages.

More and more new types of miRNAs are being identified, but few are relevant to CRC, and reports of differentially expressed miRNAs in CRC have not been well validated by other studies. All of these issues affect the better understanding of the roles of miRNAs in CRC pathogenesis and the potential of miRNAs as diagnostic markers for detection of CRC. To this end, in the present study we performed a profiling of 1547 distinct human miRNAs found in paired tumor and adjacent normal mucosa obtained from 31 CRC patients.

## Materials and Methods

### Patients and specimens

The Clinical Research Ethics Committee of Third Affiliated Hospital of Guangzhou Medical University approved the research protocols, and participants provided written informed consent. Demographic information was obtained from patient records and registries.

Sixty-two tissue specimens, including 31 tumor tissue and 31 paired adjacent normal mucosa were selected from 31 cases of CRC, comprising 15 cases of stage II, 13 of stage III, and 3 of stage IV (Table 1). Among them, 19 cases were colon cancer, and the remaining 12 were rectal cancer. All of these CRC patients received surgery at Third Affiliated Hospital of Guangzhou Medical University from June 2010 to October 2012. The majority of selected cases were stage II and stage III, because 70% of CRC patients seen in clinic are in either of these two stages [3]. In addition, 10 CRC and paired normal samples were collected from November 2013 to December 2013 for an independent sample test to confirm the previous microRNA profiling results.

**Table 1:** Clinical and histopathological characteristics of patients.

Patient ID	Age (y)	Gender	Stage	Differentiation	Anatomic site
1	72	Male	III	Moderate	Colon
2	71	Male	III	Moderate	Rectum
3	45	Male	III	Moderate	Colon
4	56	Male	II	Moderate	Rectum
5	74	Female	III	High	Colon
6	83	Female	III	Moderate	Rectum
7	38	Male	III	Moderate	Rectum
8	73	Female	IV	Poor	Colon
9	20	Female	III	Moderate	Colon
10	73	Male	II	Moderate	Colon
11	77	Female	II	Moderate	Colon
12	61	Male	II	Moderate	Rectum
13	60	Female	II	Moderate	Colon
14	60	Male	IV	Moderate	Rectum
15	72	Female	II	-*	Rectum
16	38	Male	II	High	Colon
17	71	Female	II	-*	Colon
18	60	Male	III	Poor	Rectum
19	74	Female	III	Moderate	Rectum
20	53	Female	II	Moderate	Rectum
21	77	Male	II	Moderate	Colon
22	77	Female	III	-*	Colon
23	73	Female	II	High	Colon
24	57	Female	II	Moderate	Colon
25	70	Female	II	High	Colon
26	50	Male	IV	Moderate	Colon
27	54	Female	III	Moderate	Colon
28	77	Male	II	Moderate	Rectum
29	74	Female	III	Moderate	Rectum
30	82	Male	II	High	Colon
31	82	Male	III	Poor	Colon

* Information not available.

Patients were excluded from the study if they had received preoperative chemotherapy or radiation therapy, or had a previous history of malignant tumors. All patients underwent surgical resection at the Department of Pathology, Third Affiliated Hospital of Guangzhou Medical University, with a final pathological diagnosis of CRC. All the tumor specimens were histologically classified and staged according to the seventh edition of the tumor-node-metastasis (TNM) staging system. Tissue samples were flash-frozen in liquid nitrogen after resection, and stored at −80°C until nucleic acids were extracted.

### RNA isolation

Frozen tissues (80–100 mg) were used to isolate miRNA using RNAzol reagent (Molecular Research Center), in accordance with the manufacturer's instructions. In brief, 100 mg of tissue was homogenized with 1 mL RNAzol. To the homogenate, 0.4 mL water was added. After 5–10 min, the mixture was centrifuged at 12 000× *g* for 15 min for DNA/protein precipitation. One milliliter of the supernatant was mixed with 0.4 mL of 75% ethanol for 10 min and centrifuged at 12 000× *g* for 8 min for miRNA precipitation. The collected miRNA supernatant was mixed with isopropanol (0.8 volumes) for 30 min and then centrifuged at 12 000× *g* for 15 min, washed 2× with 0.4 mL 70% isopropanol, and centrifuged at 8000× *g* for 3 min. Finally, miRNAs were dissolved in diethylpyrocarbonate-treated water.

The concentration and purity of the miRNAs were determined by electrophoresis and a NanoDrop spectrophotometer (Thermo Scientific, USA). The quality of miRNAs was considered to meet the requirements if the OD260/OD280 was between 1.7 and 2.0. The integrity of small RNAs was evaluated by the determination of robust amplification of small nuclear ubiquitous RNAs (i.e., RNU6b, RNU44, and RNU48) by real-time reverse-transcription PCR (qRT-PCR), because they are commonly used as endogenous controls in miRNA studies.

### MiRNA profiling

A Universal RT microRNA PCR system (GeneCopoeia, USA) was applied for miRNA profiling using 2 pooled tissue samples, i.e., 31 tumors and 31 paired normal controls. The assay for the profiling included a universal reverse transcription (RT) and sequential qRT-PCR amplification with special primers using SYBR Green.

### Reaction

The PCR reaction was performed in a 384-well PCR plate with each well containing 20 µL of reaction system, including 1 µL cDNA and 1 µL gene-specific PCR primers. A total volume of 50 mL PCR reaction system including 2.5 mL cDNA of each tissue sample for 9× 384-well plates was picked out and uniformly mixed. A total of 1547 distinct miRNAs were used for the profiling, and fold changes in the expressions of each type of miRNA were determined.

### Quantification of miRNAs by qRT-PCR

MiRNAs (approximately 500 ng) were reverse transcribed in 25-µL reaction volumes using an All-in-One First-Strand cDNA Synthesis kit (GeneCopoeia, USA). In short, 25 µL of RT reaction mix included the miRNA sample, 5 µL of 5× reaction buffer, 2.5 U/µL PolyA Polymerase, 10 ng/µL MS2 RNA, and RTase Mix. The reaction was performed at 37°C for 60 min, and terminated at 85°C for 5 min.

Ten-fold diluted cDNA that was produced in the RT reaction was used as templates for the PCR reaction in an Applied Biosystems ViiA 7 Real-Time PCR System (Life Technologies, USA), in which MS2 RNA was used as an external reference for the quality of extracted miRNAs, and RNU6B, RNU44, RNU48, and RNU49 were used for normalization. The PCR system contained the following components within a total 20-µL volume per well: 10 µL 2× All-in-one qPCR Mix, 2 µL PCR forward primer (2 uM), 2 µL PCR reverse primer (2 uM), 1 µL templates, 0.2 µL 50× ROX Reference Dye (for calibration) and 4.8 µL ddH_2_O. A master of the mixture was often prepared including all the components except for the template. If the total volume of the master mixture changed, each component was brought to the proper proportion. The conditions of real-time PCR were: hot-start denaturating at 95°C for 10 min; 40 cycles of amplification with denaturation at 95°C for 10 s, annealing at 60°C for 20 s, and extension at 72°C for 15 s; then melting at 60°C for 10 min and finally cooling at 25°C for 30 s.

To optimize PCR conditions, a preliminary experiment was performed to ensure that the difference in the cycle threshold (CT) value of MS2 RNA between cancer and paired normal samples did not exceed 1.0. The expression levels of the miRNAs were quantified using SYBR Green-based All-in-One qPCR Mix (GeneCopoeia, USA).

To confirm microRNA profiling results, we performed an independent sample test to compare the expression levels of 6 altered miRNAs (miR-145*, -30e*, -378*, -125a-5p, -3195, and -4770) between tumor and paired normal tissues. We chose these miRNAs specifically because we had found them to be dysregulated, but they have never been reported previously.

### Statistical analyses

Graph Pad Prism 5.0 (San Diego, CA, USA) and SPSS 16.0 (SPSS, UK) software were used for data analyses. The comparative expression of miRNAs was determined using the 2^−ΔΔCT^ method [13]. The differentially expressed miRNAs of tumor tissues relative to paired normal tissues were identified using non-paired and paired *t*-tests, as well as receiver operating characteristic (ROC) curves. The area under the ROC curve (AUC) was used for the evaluation of sensitivity and specificity of miRNAs from tissue specimens as a diagnostic marker for detection of CRC. The analysis of correlation was performed with Pearson's test. In the two-tailed test, a *P*-value<0.05 was considered statistically significant.

## Results

### Differential expressed miRNAs between tumor and paired adjacent normal mucosa

**Figure 1:**
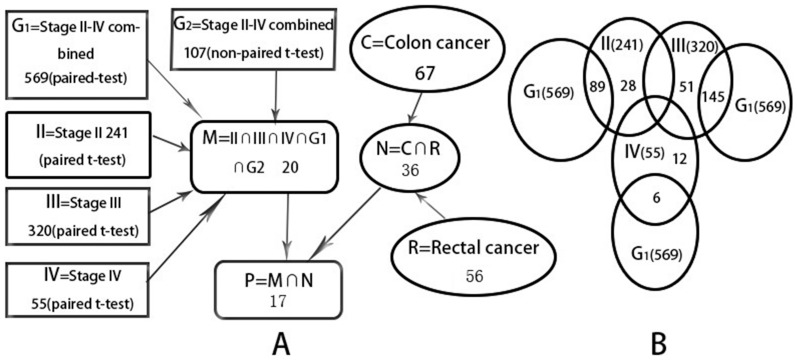
The intersection among results in our study. According to the TNM staging and origin of tissues, CRC patients were divided into stages II, III, or IV colon or rectal cancer. There were 17 dysregulated miRNAs with a similar expression pattern in all 7 groups (II, III, IV, G1, G2, C, and R); 28 dysregulated miRNAs were found only in stage II and 51 only in stage III, but stage IV had 12 dysregulated miRNAs.

According to the paired *t*-test (*P*<0.05), 569 dysregulated miRNAs were identified in 31 adenocarcinoma samples, in which 526 were downregulated and 43 were upregulated (Figure 1; Table S1).

We also performed an analysis using a non-paired *t*-test that treats normal and tumor samples as two independent groups. From that analysis, 107 significantly dysregulated miRNAs were identified, 95 of which were downregulated and 12 were upregulated (Table S2). There was an overlap in the results of the paired and non-paired analyses, but the dysregulated miRNAs identified by the non-paired *t*-test were included in the list found by the paired *t*-test. Thus, 107 dysregulated miRNAs were identified in both the paired and non-paired *t*-test, but 462 dysregulated miRNAs were identified only by the paired *t*-test. Except for miR-150, with a −1.77-fold change relative to the normal controls, the fold changes of the other 106 dysregulated miRNAs were greater than 2-fold (Table S2). The expression patterns of the dysregulated miRNAs were similar in the two analytical approaches. Among the dysregulated miRNAs, the fold changes of miR-1 and miR-145 were greatest (>15-fold). Others with fold changes greater than 6-fold included miR-145*, -137, -133a, -4470, -143, -163, and miR-490-5p. In the upregulated miRNAs, the fold changes of miR-96, miR-135b, and miR-141 were 6, 4.4, and 4.8, respectively.

### Correlation of dysregulated miRNAs and clinical characteristics

We investigated the expression patterns of the dysregulated miRNAs in each stage. Patients selected in this study were divided into stage II, stage III, and stage IV groups, the most advanced lesions we considered. Firstly, the analysis identified, via the paired *t*-test, 241 miRNAs in stage II tumors significantly dysregulated relative to the paired normal tissues. These consisted of 206 downregulated miRNAs, in which miR-145 had the highest fold-change, −18.15-fold (Table S3).

Secondly, 320 significantly dysregulated miRNAs were identified in stage III tumors, among which there were 282 downregulated miRNAs, including miR-145* with a −18.9-fold change. Other downregulated miRNAs with a fold change greater than 5-fold included miR-1, -145, -27b, -137, -144*, let-7c, miR-4469, -363, -4510, -23b, -4475, -143, -490-5p, -24-1*, -4770, -129*, -9, and -4423-3p. In 38 upregulated miRNAs, the fold change in miR-1290 expression in tumor tissues was the highest (17.7-fold), and the expressions of miR-362-5p, -367, -593, -545, -524-5p, -1246, -96, -224, -450a, -7, and -203 were greater than 5-fold (Table S4).

In stage IV tumors, 55 significantly dysregulated miRNAs were identified, in which 46 were downregulated and 9 were upregulated. Among the downregulated, miR-145* had the highest fold change (23.8-fold), while others that were downregulated more than 5-fold included miR-145, -101*, -133a, -214, -4768-3p, -4770, let-7e, miR-378*, -99a, -193b, -100, and -1185. Of the 9 upregulated miRNAs, miR-135b had the highest fold change (17.7-fold), but only 3 miRNAs (miR-200a, -429, and miR-135b) reached a change greater than 5-fold (Table S5).

Furthermore, we investigated the overlap among the results from a 4-paired *t*-test. Altogether, 23 overlapped miRNAs in the paired t-tests were found, all downregulated, including miR-145*, -145, -101*, -133a, -214, -4770, -378*, -99a, -193b, -100, -125b, -3195, -30e*, -9, -29b-2*, -125a-5p, let-7b, miR-24-1*, -27b*, -30a, -1979, -140-3p, and -768-3p. We made a comparison of the overlap between those 23 miRNAs and all (107) the dysregulated miRNAs mentioned above. There were 22 miRNAs that overlapped in the two statistical approaches, but miR-101* was only identified in the paired *t*-test. The 22 overlapping miRNAs were miR-145*, -145, -133a, -214, -4770, -378*, -99a, -193b, -100, -125b, -3195, -30e*, -9, -29b-2*, -125a-5p, let-7b, miR-24-1*, -27b*, -30a, -1979, -140-3p, and -768-3p, all of which were downregulated.

### Diagnostic value of the dysregulated miRNAs

Our findings, as mentioned above, indicated that 22 dysregulated miRNAs (miR-145*, -145, -133a, -214, -4770, -378*, -99a, -193b, -100, -125b, -3195, -30e*, -9, -29b-2*, -125a-5p, let-7b, -24-1*, -27b*, -30a, -1979, -140-3p, and -768-3p) had good potential as biomarkers for distinguishing CRC patients from normal individuals. To verify this possibility, we performed an ROC analysis in terms of the normalized cycle threshold values (ΔCt) of miRNAs.

**Figure 2:**
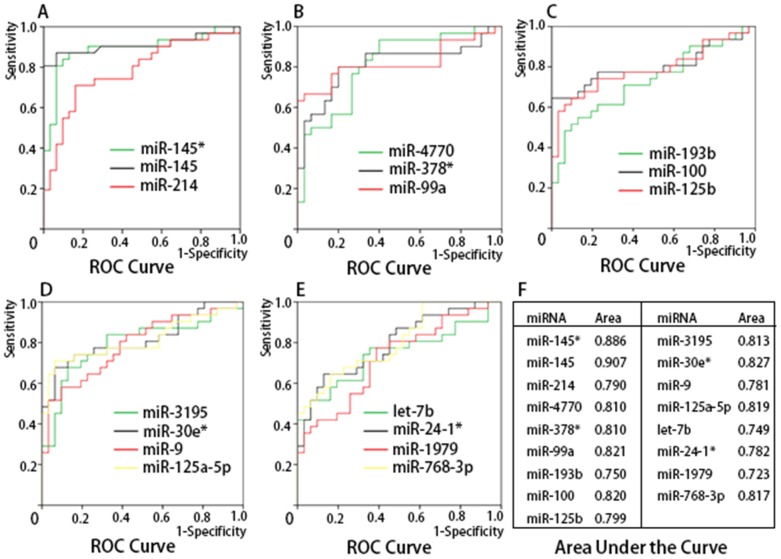
ROC curve analysis of 17 dysregulated miRNAs. Analysis of data implied that most of the dysregulated miRNAs have potential as diagnostic biomarkers for CRC detection, with high sensitivity and specificity.

The expressions of 17 of the dysregulated miRNAs (miR-145*, -145, -214, -4770, -378*, -99a, -193b, -100, -125b, -3195, -30e*, -9, -125a-5p, let-7b, miR-24-1*, -1979, and -768-3p) were significantly lower in both colon and rectal cancers compared with normal tissues, but of the remaining 5, miR-133a and miR-140-3p were found significantly downregulated (*P*<0.05) only in rectal cancers, and miR-27b*, miR-30a, and miR-29b-2* were significantly downregulated only in colon cancers (*P*<0.05; Figure 1). Therefore, ROC curves were plotted for the 17 dysregulated miRNAs in both colon and rectal cancers for the validation of a cut-off value that can distinguish CRC patients from the normal population (Figure 2).

To confirm the reproducibility of our findings, we performed an independent sample test for expression levels of miR-145*,-30e*,-378*,-125a-5p,-3195 and -4770. The result of this test showed that the above mentioned 6 miRNAs are downregulated in CRC tumor, consistent with our previous findings (Table 2).

**Table 2:** Expression levels of miR-145*, -30e*, -378*, -125a-5p, -3195, and -4770 in an independent sample test.

	Fold change	*P*-value
miR-145*	−18.1818	0.0000
miR-30e*	−41.6667	0.0000
miR-378*	−10.7527	0.0000
miR-125a-5p	−111.1111	0.0000
miR-3195	−7.0423	0.0000
miR-4770	−8.0000	0.0000

### Novel biomarkers in CRC without metastasis

It has been reported that the prognosis of cancer patients closely correlates with the stage of the tumor, and more than half of CRC patients have lymph node or distant metastasis at the time of diagnosis [14]. To address this issue, we tried to find miRNAs that may serve as biomarkers especially for identification of CRC patients without lymph node or distant metastasis. We focused on those miRNAs that were aberrantly expressed in stage II tumors (*P*<0.05), rather than in other stages. Based on this criterion, among 241 dysregulated miRNAs there were 35 upregulated miRNAs and 206 downregulated miRNAs. Of these, 89 were found in both stage II and stage II–IV tumors. Only 28 miRNAs were involved in stage II tumors, in which 8 were upregulated (miR-1274a, -200c, -148a, -532-5p, -19b, -503, -320c, and -1261) and the rest were downregulated (miR-3647-5p, -374a, -2054, -1323, -4795-5p, -4801, -3199, -190, -923, -29c, -4804-5p, -454, -4320, -551b, -4764-5p, -4763-3p, -449b*, -4797-3p, -186, and -371-5p).

**Figure 3:**
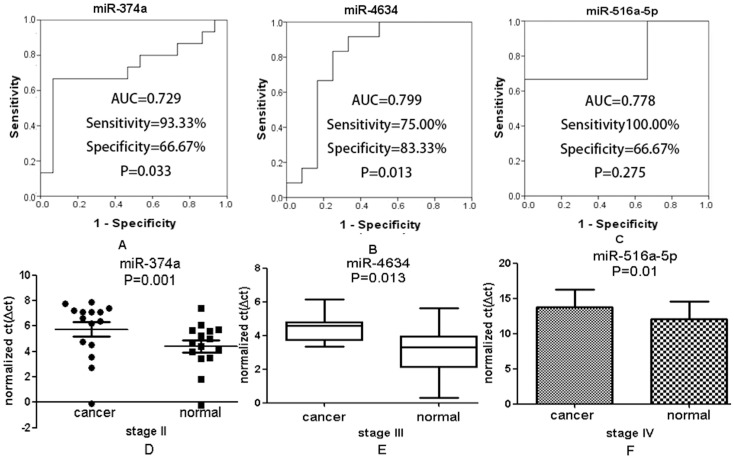
Evaluation of the potential of miR-374a, miR-4634, miR-516a-5p as diagnostic biomarkers in clinical application. Among them, miR-374a was dysregulated in stage II, miR-4634 was related to lymph node metastasis in stage III, and miR-516a-5p might be associated with distant metastasis.

We also evaluated the accuracy of miR-374a as a diagnostic marker for identification of CRC patients without metastasis, because miR-374a expression was significantly decreased in stage II (*P* = 0.001; Figure 3). Through ROC curve analysis, the effectiveness of miR-374a as a diagnostic marker was confirmed by the following results: AUC 0.729, sensitivity 93.33%, specificity 66.67%, and *P* = 0.033.

### Predictive value of miRNAs for CRC patients with lymph node and distant metastasis

To further investigate the prognostic value of miRNAs for CRC detection, we focused on the dysregulated miRNAs that were only found in stage III, a stage with lymph node metastasis but without distant metastasis. Among 320 differentially expressed miRNAs found in stage III, only 51 were expressed uniquely in stage III, including 22 upregulated and 29 downregulated (Figure 1). The expression of miR-4634 was significantly decreased in stage III (*P* = 0.013), which has never been reported previously. ROC analysis further supported the potential of miR-4634 as a biomarker for detection of lymph node metastasis, with the following results: AUC 0.799, sensitivity 75.00%, specificity 83.33%, and *P* = 0.013 (Figure 3).

We found 55 miRNAs aberrantly expressed in stage IV, a stage that includes distant metastasis. The number of dysregulated miRNAs found uniquely in stage IV (12 miRNAs, Figure 1) were fewer than the number of miRNAs unique to other stages. In those 12 miRNAs, 4 were upregulated (miR-194, -449b, -425, and –3153), and 8 were downregulated. To explore their potential as diagnostic markers, we selected one of them, miR-516a-5p, for ROC analysis (Figure 3). Based on the *P*-value (*P* = 0.275), the ROC analysis indicated that miR-516a-5p is not an ideal biomarker that can distinguish CRC patients in stage IV from normal individuals, although other parameters of the ROC analysis were attractive: AUC 0.778, sensitivity 100.0%, specificity 66.67%, and *P* = 0.033. However, it might be too early to make conclusions regarding miR-516a-5p, due to limitations in our study caused by small samples. Therefore, further study with larger samples for greater statistical power is warranted.

## Discussion

In the present study, we performed a miRNA profiling of 31 pairs of tumor and normal mucosa obtained from 31 CRC patients, and identified 569 miRNAs that were significantly dysregulated in CRC relative to normal tissues (*P*<0.05). Among the 569 dysregulated miRNAs, 17 downregulated miRNAs were associated with separate or combined stages II, III, and IV colon and rectal cancers. ROC curve analysis indicated that most of the 17 downregulated miRNAs had potential as useful biomarkers with high sensitivity and specificity for CRC detection. Furthermore, we found that several dysregulated miRNAs may serve as biomarkers especially for identification of CRC patients without lymph node or distant metastasis. The value of miRNAs for CRC patients with lymph node and distant metastasis was also evaluated in our study.

**Figure 4:**
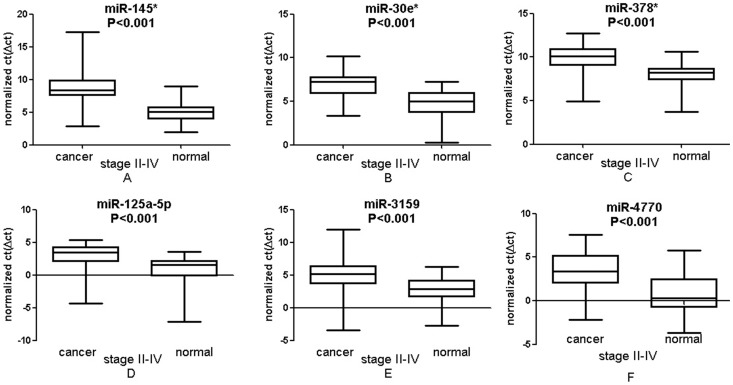
Expression levels of dysregulated miRNAs. Box plots of expression levels of six miRNAs in both tumors and paired normal tissues. The relative level of the six miRNAs was normalized to internal control gene and was showed as normalized CT. The line represents the median value—the higher the value, the lower the expression level. Paired t-tests were performed to examine the differences in miRNAs between tumors and the paired normal tissues.

Among 17 identified miRNAs, some have been reported previously, such as miR-145 and miR-214, consistent with our findings [15], [16]. However, in contrast to our findings, miR-9 was reported to be upregulated in Zhu et al.'s [17] study. The difference between the two studies may be due to the diversity of techniques and samples used. Most importantly, several identified miRNAs in our study, such as miR-145*, -30e*, -378*, -125a-5p, -3195, and -4770, have been rarely mentioned in the literature. Thus, we are the first to demonstrate the differential expression patterns of these miRNAs in cancer tissues relative to normal tissues (Figure 4). The pattern of altered miRNAs was confirmed by an independent sample test that verified the expression levels of miR-145*,-30e*,-378*,-125a-5p,-3195 and -4770. The results of the independent sample test are consistent with the miRNA profiling data, indicating that our conclusion is reliable and reproducible (Table 2).

Moreover, we evaluated via ROC analysis the potential of identified miRNAs as diagnostic biomarkers in CRC without metastasis, and their value for predicting lymph node or distant metastasis. Their expression patterns in either colon or rectal cancers were also examined.

Recently, aberrant expression of miRNAs has been implicated in numerous diseases, including human carcinomas [18]. For example, downregulation of miRNAs, such as miR-145, -195, -383 and miR-378, was found in CRC relative to their expressions in normal mucosa, whereas, some upregulated miRNAs, like miR-96, -135b, miR-493 and miR-133a, have also been found associated with CRC [19], [20].

Moreover, the mechanisms and molecular pathways related to the roles of miRNAs in carcinogenesis have become hot spots of miRNA studies. Significant progress in this field has been made, for example, Ma et al. [21] reported that miR-17-5p is involved in tumorigenesis and tumor progression; this oncogenic miRNA targets the gene encoding P130, leading to activation of the Wnt/β-catenin pathway. Another example is a study that showed that miR-218 can suppress expression of cyclin-dependent kinase 4 (CDK4), a BMI1 downstream target, but increased p53 expression [22]. Furthermore, it is reported that miR-31 contributes to the development of colon cancer at least partly by targeting RhoBTB1 (Rho-related BTB domain containing 1) [23].

However, previously reported dysregulated miRNAs in CRC have not been well verified by further studies. The results reported in several papers are inconsistent and controversial. Schee and colleagues [24] reported that in a patient cohort, further investigation of miRNAs previously identified as candidate biomarkers in CRC (miR-21, -31, -92a, -101, -106a, -145) was not successful; the main reason was lack of clinically relevant associations. Chen et al. [25] also pointed out that results regarding aberrant miRNAs found in their study were not consistent with previous reports.

To address this issue, our study was designed to identify alterations in global miRNA expressions between CRC tumors and paired normal tissues. From analyses using the paired *t*-test we found 569 aberrantly expressed miRNAs in CRC tumors (*P*<0.05; Table S1). Among them, there are 526 downregulated and 43 upregulated miRNAs. The majority of dysregulated miRNAs in CRC were downregulated, but a few were upregulated. These findings are consistent with previous studies [6], [26], in that the role of the majority of miRNAs in CRC cells is that of growth suppressors [3]. Since the non-paired *t*-test is more conservative compared with the paired *t*-test, we identified the aberrant expressions of miRNAs in CRC tumors relative to normal tissues based on the results of the non-paired *t*-test [19]. Through this statistical approach, 107 dysregulated miRNAs were found. All of these were also identified by the paired *t*-test. These miRNAs might have potential as novel biomarkers for CRC detection in the future.

To meet the requirement as a biomarker for CRC detection, the screening test for dysregulated miRNAs should be able to distinguish patients with either colon or rectal cancer, in every stage, from healthy persons. Otherwise, a misdiagnosis or missed diagnosis may occur. Therefore, we further investigated the expression patterns of miRNAs in each stage II–IV, either for colon or rectal cancer, through a cross-validation approach. Altogether, 17 dysregulated miRNAs that have similar expression patterns in both colon and rectal cancer were identified, including miR-145*, -145, -214, -4770, -378*, -99a, -193b, -100, -125b, -3195, -30e*, -9, -125a-5p, let-7b, miR-24-1*, -1979, and -768-3p. The involvement of some of these miRNAs in CRC has recently been reported, but the others have not been. Our findings are supported by some previous studies, but several contradictions or inconsistencies remain regarding the function of some miRNAs as described elsewhere. For example, the expression pattern of miR-145 reported in Hamfjord et al.'s [19] study is the same as our findings, but that of miR-7 differs. Another example is that expressions of miR-23a and miR-10a were found to be decreased in Xi et al.'s [27] study consistent with our findings, but expressions of miR-21 and -27b were the opposite.

Such inconsistencies may be due to differences in patient ethnicities, locations, genes, or screening criteria, or any combination of these factors, that could be resolved by establishing standards for screening in the near future [23]. Differences in research techniques may also account for such inconsistencies, and thus cost effective, simple, and reliable techniques are desirable in clinical application. Finally, differences in results may be due to faulty experimental designs that should be improved in future studies.

The present study is limited by the small number of samples (in total, 31), especially of samples of stage I and stage IV tumors. These are comparatively rare in local hospitals in Guangdong province. Therefore, the findings of the present study need further validation in a larger cohort of CRC patients. Despite this disadvantage, by a statistical approach using both paired and non-paired tests our study initially found 17 downregulated miRNAs in stages II, III, and IV colon and rectal cancers. We also explored dysregulated miRNAs for potential as diagnostic biomarkers for CRC patients without metastasis, as well as their value to predict lymph node and distant metastasis in CRC. Whether these miRNAs will be confirmed as CRC diagnostic biomarkers in future clinical trials remains a question, but further investigations are warranted.

Although our findings are not immediately applicable in clinical practice, the detection of dysregulated miRNAs that we have described herein is a novel and viable approach for the detection of human CRC.

## Supporting Information

Table S1 **Dysregulated miRNAs in CRC tissues compared with normal group with paired t-test.**
(XLSX)

Table S2 **Dysregulated miRNAs in CRC tissues compared with normal group with non-paired t-test.**
(XLSX)

Table S3 **Dysregulated miRNAs in CRC tissues compared with normal group in stage II with paired t-test.**
(XLSX)

Table S4 **Dysregulated miRNAs in CRC tissues compared with normal group in stage III with paired t-test.**
(XLSX)

Table S5 **Dysregulated miRNAs in CRC tissues compared with normal group in stage IV with paired t-test.**
(XLSX)

## References

[1] Bandres E, Cubedo E, Agirre X, Malumbres R, Zarate R, et al (2006) Identification by Real-time PCR of 13 mature microRNAs differentially expressed in colorectal cancer and non-tumoral tissues. Mol Cancer 5: 29. 16854228 10.1186/1476-4598-5-29 PMC1550420

[2] Kuo TY, Hsi E, Yang IP, Tsai PC, Wang JY, et al (2012) Computational analysis of mRNA expression profiles identifies microRNA-29a/c as predictor of colorectal cancer early recurrence. PLoS One 7: e31587. 22348113 10.1371/journal.pone.0031587 PMC3278467

[3] Suzuki H, Takatsuka S, Akashi H, Yamamoto E, Nojima M, et al (2011) Genome-wide profiling of chromatin signatures reveals epigenetic regulation of MicroRNA genes in colorectal cancer. Cancer Res 71: 5646–5658. 21734013 10.1158/0008-5472.CAN-11-1076

[4] Lee RC, Feinbaum RL, Ambros V (1993) The C. elegans heterochronic gene lin-4 encodes small RNAs with antisense complementarity to lin-14. Cell 75: 843–854. 8252621 10.1016/0092-8674(93)90529-y

[5] Lewis BP, Burge CB, Bartel DP (2005) Conserved seed pairing, often flanked by adenosines, indicates that thousands of human genes are microRNA targets. Cell 120: 15–20. 15652477 10.1016/j.cell.2004.12.035

[6] Lu J, Getz G, Miska EA, Alvarez-Saavedra E, Lamb J, et al (2005) MicroRNA expression profiles classify human cancers. Nature 435: 834–838. 15944708 10.1038/nature03702

[7] Calin GA, Croce CM (2006) MicroRNA signatures in human cancers. Nat Rev Cancer 6: 857–866. 17060945 10.1038/nrc1997

[8] Kong YW, Ferland-McCollough D, Jackson TJ, Bushell M (2012) microRNAs in cancer management. Lancet Oncol 13: e249–258. 22652233 10.1016/S1470-2045(12)70073-6

[9] Calin GA, Sevignani C, Dumitru CD, Hyslop T, Noch E, et al (2004) Human microRNA genes are frequently located at fragile sites and genomic regions involved in cancers. Proc Natl Acad Sci U S A 101: 2999–3004. 14973191 10.1073/pnas.0307323101 PMC365734

[10] Piepoli A, Tavano F, Copetti M, Mazza T, Palumbo O, et al (2012) Mirna expression profiles identify drivers in colorectal and pancreatic cancers. PLoS One 7: e33663. 22479426 10.1371/journal.pone.0033663 PMC3316496

[11] Schimanski CC, Frerichs K, Rahman F, Berger M, Lang H, et al (2009) High miR-196a levels promote the oncogenic phenotype of colorectal cancer cells. World J Gastroenterol 15: 2089–2096. 19418581 10.3748/wjg.15.2089 PMC2678579

[12] Wang Q, Huang Z, Ni S, Xiao X, Xu Q, et al (2012) Plasma miR-601 and miR-760 are novel biomarkers for the early detection of colorectal cancer. PLoS One 7: e44398. 22970209 10.1371/journal.pone.0044398 PMC3435315

[13] Livak KJ, Schmittgen TD (2001) Analysis of relative gene expression data using real-time quantitative PCR and the 2(-Delta Delta C(T)) Method. Methods 25: 402–408. 11846609 10.1006/meth.2001.1262

[14] Figueredo A, Coombes ME, Mukherjee S (2008) Adjuvant therapy for completely resected stage II colon cancer. Cochrane Database Syst Rev CD005390. 18646127 10.1002/14651858.CD005390.pub2 PMC8885310

[15] Yin Y, Yan ZP, Lu NN, Xu Q, He J, et al (2013) Downregulation of miR-145 associated with cancer progression and VEGF transcriptional activation by targeting N-RAS and IRS1. Biochim Biophys Acta 1829: 239–247. 23201159 10.1016/j.bbagrm.2012.11.006

[16] Xia H, Ooi LL, Hui KM (2012) MiR-214 targets beta-catenin pathway to suppress invasion, stem-like traits and recurrence of human hepatocellular carcinoma. PLoS One 7: e44206. 22962603 10.1371/journal.pone.0044206 PMC3433464

[17] Zhu L, Chen H, Zhou D, Li D, Bai R, et al (2012) MicroRNA-9 up-regulation is involved in colorectal cancer metastasis via promoting cell motility. Med Oncol 29: 1037–1043. 21562850 10.1007/s12032-011-9975-z

[18] Olaru AV, Selaru FM, Mori Y, Vazquez C, David S, et al (2011) Dynamic changes in the expression of MicroRNA-31 during inflammatory bowel disease-associated neoplastic transformation. Inflamm Bowel Dis 17: 221–231. 20848542 10.1002/ibd.21359 PMC3006011

[19] Hamfjord J, Stangeland AM, Hughes T, Skrede ML, Tveit KM, et al (2012) Differential expression of miRNAs in colorectal cancer: comparison of paired tumor tissue and adjacent normal mucosa using high-throughput sequencing. PLoS One 7: e34150. 22529906 10.1371/journal.pone.0034150 PMC3328481

[20] Sarver AL, French AJ, Borralho PM, Thayanithy V, Oberg AL, et al (2009) Human colon cancer profiles show differential microRNA expression depending on mismatch repair status and are characteristic of undifferentiated proliferative states. BMC Cancer 9: 401. 19922656 10.1186/1471-2407-9-401 PMC2787532

[21] Ma Y, Zhang P, Wang F, Zhang H, Yang Y, et al (2012) Elevated oncofoetal miR-17-5p expression regulates colorectal cancer progression by repressing its target gene P130. Nat Commun 3: 1291. 23250421 10.1038/ncomms2276

[22] He X, Dong Y, Wu CW, Zhao Z, Ng SS, et al (2012) MicroRNA-218 inhibits cell cycle progression and promotes apoptosis in colon cancer by downregulating BMI1 polycomb ring finger oncogene. Mol Med 18: 1491–1498. 10.2119/molmed.2012.00304 PMC3576472 23255074

[23] Xu RS, Wu XD, Zhang SQ, Li CF, Yang L, et al (2013) The tumor suppressor gene RhoBTB1 is a novel target of miR-31 in human colon cancer. Int J Oncol 42: 676–682. 23258531 10.3892/ijo.2012.1746

[24] Schee K, Boye K, Abrahamsen TW, Fodstad O, Flatmark K (2012) Clinical relevance of microRNA miR-21, miR-31, miR-92a, miR-101, miR-106a and miR-145 in colorectal cancer. BMC Cancer 12: 505. 23121918 10.1186/1471-2407-12-505 PMC3519622

[25] Chen WC, Lin MS, Ye YL, Gao HJ, Song ZY, et al (2012) microRNA expression pattern and its alteration following celecoxib intervention in human colorectal cancer. Exp Ther Med 3: 1039–1048. 22970014 10.3892/etm.2012.531 PMC3438602

[26] Kumar MS, Lu J, Mercer KL, Golub TR, Jacks T (2007) Impaired microRNA processing enhances cellular transformation and tumorigenesis. Nat Genet 39: 673–677. 17401365 10.1038/ng2003

[27] Xi Y, Shalgi R, Fodstad O, Pilpel Y, Ju J (2006) Differentially regulated micro-RNAs and actively translated messenger RNA transcripts by tumor suppressor p53 in colon cancer. Clin Cancer Res 12: 2014–2024. 16609010 10.1158/1078-0432.CCR-05-1853

